# Increased CSF NFL in Non-demented Parkinson’s Disease Subjects Reflects Early White Matter Damage

**DOI:** 10.3389/fnagi.2020.00128

**Published:** 2020-05-14

**Authors:** Ewa Papuć, Konrad Rejdak

**Affiliations:** Department of Neurology, Medical University of Lublin, Lublin, Poland

**Keywords:** neurofilament, Parkinson’s disease, cerebrospinal fluid, white matter damage, axonal damage

## Abstract

Parkinson’s disease (PD) is a chronic neurodegenerative disorder with various underlying pathological processes. Until now, no fluid biomarkers have been established for PD. Given recent biochemical and neuroimaging evidence for the presence of white matter damage in PD, which may even precede neuronal loss, we investigated whether neurofilament light (NFL) was increased in the cerebrospinal fluid (CSF) of PD patients in comparison to controls. NFL is located mainly in large myelinated axons, and increased CSF levels of this protein reflect axonal injury. CSF levels of NFL in 58 early PD patients and 28 controls were quantified by ELISA (Uman Diagnostics). Measures of PD severity included disease duration, UPDRS-III, and Hoehn-Yahr stage. Statistically significant differences in CSF NFL levels were found between PD patients and controls [median with interquartile range 524.82 (393.28–678.34) vs. 271.84 (198.09–335.24) ng/l; *p* < 0.05)]. In PD patients, there were no correlations between CSF NFL level and the measures of disease severity. The CSF NFL turned out to have a high discriminatory value (AUC 0.850) for differentiating between PD subjects and healthy controls, with 84% sensitivity and 85.2% specificity. The study indirectly demonstrates that axonal damage is present in early PD in addition to neuronal loss. Interestingly, white matter damage was observed in non-demented PD patients. In the light of the results of recent MRI studies which confirm early white matter damage in PD, our data may turn out to be potentially useful in the diagnosis of early, or even preclinical, stages of the disease.

## Introduction

Parkinson’s disease (PD) is a chronic neurodegenerative disorder with various underlying pathologies. Gray matter integrity loss in PD has been extensively investigated (Beyer et al., 2007; Melzer et al., 2012; Koshimori et al., 2015). In contrast, little attention has been paid so far to white matter damage in the course of the disease. Some neuroimaging studies describe white matter abnormalities in PD, but only in cognitively impaired patients (Deng et al., 2013; Agosta et al., 2014). Interestingly, data from two recent MRI studies suggest that white matter damage may precede neuronal loss in PD subjects (Agosta et al., 2013; Rektor et al., 2018).

A biomarker that indirectly reflects brain white matter damage is neurofilament light (NFL). Neurofilament (NF) proteins are highly phosphorylated proteins of the neuronal cytoskeleton. NF in the CNS is composed of four subunits (or chains): NFL, NF middle (NF-M), NF heavy (NF-H), and α-internexin (Yuan et al., 2012). NFL, which is the smallest of these NF subunits, is mainly found in large myelinated axons. Like the tau protein and microtubules, NFL is a key structural element of neurons, as it plays a role in maintaining axonal integrity, proper conduction velocity, and normal synaptic function (Fuchs and Cleveland, 1998). Upon axonal damage, NFL is released into the CSF and eventually into the blood. Elevated NFL levels are believed to correlate with more advanced axonal degeneration.

Today, cerebrospinal fluid (CSF) NFL has an established position as a biomarker in the differential diagnosis between PD and atypical parkinsonism (Abdo et al., 2007; Constantinescu et al., 2010). Elevated CSF NFL levels in atypical parkinsonism in comparison to PD reflect more widespread axonal damage (Constantinescu et al., 2010).

NFL has been considered as a marker of axonal damage in different neurological disorders (Herbert et al., 2015) and, in the light of some recent data (Backstrom et al., 2015), has become of potential interest as a CSF biomarker in PD. There is emerging evidence that NFL levels in the CSF of PD patients may be significantly increased in comparison to healthy control subjects (Oosterveld et al., 2020). Of note, abnormal aggregation of NF in the Lewy bodies of subjects with PD was described as early as 1991 (Schmidt et al., 1991). The authors of that study described the presence of neuronal inclusions rich in NF proteins in different sites of the CNS, which also reflected disruption of NFL metabolism in PD. Later human post mortem studies confirmed that the NFL is involved in Lewy body formation and that axonal transport damage precedes dopaminergic neurodegeneration in PD (Chu et al., 2012).

The results of previous studies assessing CSF NFL levels in PD patients in comparison to controls are inconsistent. While some authors have found increased levels of this biomarker in the CSF of PD patients compared to healthy controls (Backstrom et al., 2015; Oosterveld et al., 2020), many others have observed no differences between these two groups of subjects (Constantinescu et al., 2010; Herbert et al., 2015; Hansson et al., 2017).

Given this background, the present study aimed to measure CSF NFL levels in non-demented early-stage PD patients relative to healthy control subjects. In the light of recent neuroimaging data (Rektor et al., 2018), CSF NFL could be potentially of interest in the diagnosis of the preclinical stage of PD, if axonal damage does precede gray matter deterioration.

## Materials and Methods

Fifty-eight patients with early PD and 28 healthy controls were enrolled in the study. PD patients in the early stage of the disease (scores of 1–3 on the Hoehn-Yahr scale) who were admitted to the Department of Neurology of the Medical University of Lublin, Poland, were enrolled. All PD patients who gave their informed consent were assessed on the UPDRS (Fahn and Elton, 1987) and H-Y (Jankovic et al., 1990) and had a lumbar puncture performed by one neurologist specialized in movement disorders. The patients’ cognitive status was assessed using MMSE. PD patients with dementia were excluded from the study.

CSF was obtained by lumbar puncture at L3–L4 or L4–L5 and collected in polypropylene tubes. CSF was routinely assessed for cell count and protein and glucose levels, after which it was centrifuged at 1,800 g for 10 min, and stored at −80°C within 2 h from the lumbar puncture, in compliance with the current guidelines (Teunissen et al., 2009).

CSF NFL levels were measured using a commercially available ELISA kit, following the manufacturer’s instructions (Uman Diagnostics; Petzold et al., 2010). Clinical data of the subjects who underwent CSF assessment were blinded to laboratory analysis. NFL levels were estimated and expressed in nanograms per liter (ng/l). All samples were analyzed in duplicate.

Also, CSF samples from 28 age-matched (*p* > 0.05) and gender-matched healthy controls were collected. The control subjects were in-patients at the Department of Neurology, who required lumbar puncture mainly due to severe headache, to exclude subarachnoid hemorrhage or meningitis and had a pathological process in the CNS excluded.

The study was approved by the local ethics committee of the Medical University of Lublin, Poland (consent ID KE-0254/292/2017), and all study participants gave written informed consent for participation in the study.

### Statistical Analysis

Data with normal distribution were presented as means with standard deviation (SD); the minimum and maximum values were also shown. Because CSF NFL values were not normally distributed, the data for CSF NFL were presented as medians with interquartile ranges. Chi-square test and student’s *t*-test were used, respectively, to compare gender and age between PD patients and controls. For comparisons of CSF NFL levels between groups, the U Mann–Whitney test was performed.

The correlations between CSF NFL and age, disease severity, disease duration, and levodopa dose were assessed using rho Spearman correlation coefficients.

The diagnostic accuracy of CSF NFL in differentiating between PD and healthy subjects was calculated based on the area under the curve (AUC) of the receiver operating characteristic curve.

Cut-off values were calculated using Youden’s index, which maximizes the sum of sensitivity and specificity.

Statistical significance was set at *p* < 0.05. Statistical analysis was performed using Graph-Pad.

## Results

**Figure 1:**
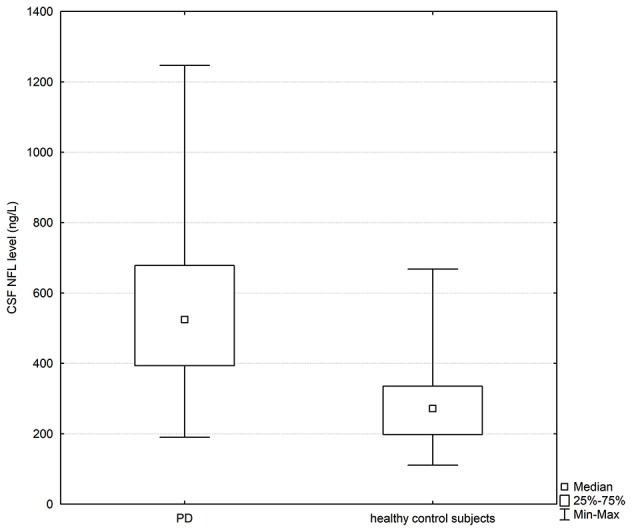
Box plot of cerebrospinal fluid (CSF) neurofilament light (NFL) levels in PD subjects and healthy controls. The central square point in each box indicates the median, box edges mark the first and third quartiles, and limits of the vertical lines show ranges. PD, Parkinson’s disease.

The PD and healthy control groups were matched for gender and age, and no statistically significant differences between the investigated groups were found in terms of age (*p* > 0.05). Clinical and biochemical characteristics of the study population is presented in Table 1. Statistically significant differences in CSF NFL levels were observed between PD patients and healthy controls; CSF NFL data are presented as median values with interquartile range [524.82 (393.28–678.34) vs. 271.84 (198.09–335.24) ng/l; *p* < 0.05; Figure 1].

**Table 1:** Clinical and biochemical characteristics of the study population.

	Parkinson’s disease patients (*n* = 58)	Healthy controls (*n* = 28)
Age (years), mean ± SD (Range)	65.18 ± 8.54 (50–83)	63.14 ± 13.71 (50–77)
Gender (M/F)	41/17	20/8
MMSE (mean ± SD)	26.46 ± 2.46	28.86 ± 1.11
Pure L-dopa daily dose (mg)	332.14 ± 125.81	NA
L-dopa daily equivalent (mg)	527.14 ± 205.75	NA
Hoehn-Yahr scale	2.3 ± 0.89	NA
UPDRS III “ON”	17.57 ± 7.60	NA
UPDRS III “OFF”	31.57 ± 10.00	NA
Disease duration (years)	2.61 ± 2.15	NA
CSF NFL level (ng/l); median with interquartile range	524.82 (393.28–678.34)	271.84 (198.09–335.24)

*MMSE, Mini-Mental State Examination; NA, not applicable. For normally distributed values, data are presented as means with standard deviation (SD). CSF NFL levels (not normally distributed) are shown as median values with interquartile ranges*.

In PD patients, no correlation was found between CSF NFL levels and disease severity assessed on the H-Y scale (*p* = 0.943) or UPDRS part III (*p* = 0.951).

Also, no correlations were found between CSF NFL levels and disease duration (*p* = 0.937), overall levodopa dose (*p* = 0.557), or levodopa equivalent (*p* = 0.735).

In the group of PD patients, there was a significant positive correlation between CSF NFL levels and age (*R* = 0.64, *p* < 0.0001). Interestingly, this correlation was not observed in healthy controls. Correlation coefficients between CSF NFL and clinical variables are presented in the Table 2.

**Table 2:** Rho Spearman correlation coefficients between CSF NFL levels and clinical variables (stage of the PD on Hoehn-Yahr scale, UPDRS “ON” state, UPDRS “OFF” state and disease duration) in PD patients.

Clinical variable	CSF NFL level (ng/l)	*p*
Hoehn-Yahr scale	0.480514	*P* = 0.009649
UPDRS “ON”	−0.101138	*p* > 0.05
UPDRS “OFF”	−0.003293	*p* > 0.05
PD duration	0.049075	*p* > 0.05

**Figure 2:**
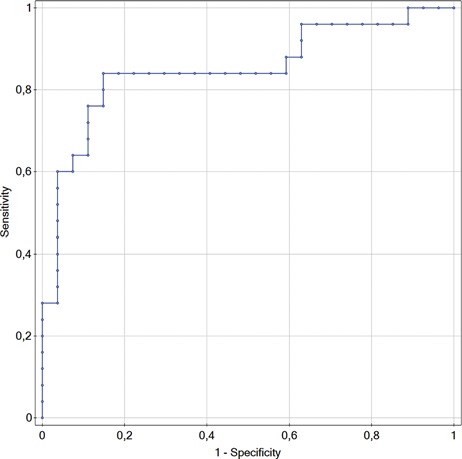
Roc curve and area under the curve (AUC) for CSF NFL in PD.

The CSF NFL chain alone provided a high discrimination (AUC 0.850) between PD subjects and healthy controls, with 84% sensitivity and 85.2% specificity at a cut-off value of 350.66 ng/l (Figure 2).

## Discussion

The fact that there exist established CSF biomarkers for Alzheimer’s disease (Tau, p-tau, Aβ-42) spurs interest in finding a specific CSF biomarker for PD to support the clinical diagnosis of this disease. Many studies have focused on the quantification of α-synuclein in CSF of patients with parkinsonian syndromes, but the results preclude its use as a biomarker of PD (Kang et al., 2013; Parnetti et al., 2013).

CSF NFL allows to differentiate PD from atypical parkinsonisms with high specificity, sensitivity and accuracy (Constantinescu et al., 2010; Herbert et al., 2015; Rektor et al., 2018), but the results of studies on the applicability of CSF NFL assessment in the differential diagnosis between PD and healthy subjects are inconsistent. Some previous found no differences in CSF NFL levels between PD and healthy controls, but the conclusions were based either on a smaller number of PD patients, compared to our study (Constantinescu et al., 2010; Herbert et al., 2015; Gaetani et al., 2018), or involved subjects with less advanced PD.

Our study revealed that PD patients have significantly increased CSF NFL levels compared to healthy control subjects. The results of this study indirectly suggest that in PD, the degeneration of dopaminergic neurons of the midbrain substantia nigra may be accompanied by early axonal degeneration. Importantly, our control subjects were matched for age (*p* > 0.05), as there is a high age-dependency of CSF NFL levels. Of course, we cannot exclude that the elevated CSF NFL levels reflect neuronal degeneration with the release of disrupted NFL from body cells. Normally, phosphorylated NF is localized mainly in the axons of healthy neurons. Nevertheless, in amyotrophic lateral sclerosis, another neurodegenerative disorder, pathological disruptions of the NFL, taking the form of spheroids, are localized also in proximal axons and body cells (Manetto et al., 1988; Mizusawa et al., 1989).

The reason for primary neuronal damage in PD could be the spread of α-synuclein pathology from the enteric nervous system to the brain through the vagus nerve, this concept has been presented by Braak et al. (2006). Additionally, some gut metabolites may promote microglia to the inflammatory response (Erny et al., 2015). There is evidence that microglial activation and neuroinflammation may alter neuronal function and promote neuronal death in PD (Kannarkat et al., 2013; Sanchez-Guajardo et al., 2013).

Nevertheless, presently there is emerging evidence that increased levels of NFL reflect rather white matter damage than neuronal degeneration (Petzold et al., 2010; Herbert et al., 2015). It is worth noting that our cohort consisted of non-demented PD patients only, as it is known that PD patients with MCI or with dementia may have a certain level of white matter and gray matter damage (Auning et al., 2014; Mak et al., 2015; Sterling et al., 2016). It should be noted here that axonal dysfunction occurs also in the healthy aging brain, but probably to the extent which is not reflected by a significant increase of CSF NFL. Neurodegenerative process in PD probably attenuates axonal damage and this could help explain the positive correlation of CSF NFL levels with age in a group of our PD patients.

The classical view on white matter degeneration in PD, as a phenomenon secondary to gray matter degeneration (Zhang et al., 2015; Chen et al., 2016) has been recently challenged by the results of some MRI studies which have raised the possibility that white matter damage may precede neuronal loss (Lee et al., 2010; Mak et al., 2015; Rektor et al., 2018). Recent data from neuroimaging studies, on white matter microstructure abnormalities which precede neuronal loss (Rektor et al., 2018) support the assumption that increased CSF NFL levels in PD patients reflect axonal injury or axonal loss within the CNS. Potentially, CSF NFL levels may also reflect peripheral nervous system damage with axonal injury (Mariotto et al., 2018), but none of our patients presented clinical symptoms suggestive of polyneuropathy.

NFL may be increased both in the plasma and the CSF of subjects with different neurodegenerative disorders, in which white matter damage is present (Abdo et al., 2007; Beyer et al., 2007; Hall et al., 2012; Magdalinou et al., 2015). For this reason, CSF NFL could potentially serve as a non-specific biomarker of axonal injury in different CNS disorders. Our results are in agreement with the results of some MRI studies reported by other authors which confirm the loss of white matter integrity even in early stages of PD, in subjects with normal cognition (Melzer et al., 2012; Rektor et al., 2018).

Since the NFL seems to be a non-specific marker of axonal injury in various neurological disorders, it cannot be used as a single biomarker to differentiate PD patients from controls. Nevertheless, the results of our study do show, in line with some recently published results, that increased CSF NFL reflects axonal damage in PD, which may be a primary pathological process (Backstrom et al., 2015; Oosterveld et al., 2020).

CSF NFL showed a high discriminatory value for differentiating between PD and control subjects, so its levels may be helpful in the differential diagnosis between PD and healthy subjects, especially when used with other CSF biomarkers (Parnetti et al., 2013; Oosterveld et al., 2020), as, used alone, it reflects only one pathological process present in PD.

We found no correlations between CSF NFL level in our PD patients and disease duration or disease severity assessed on the H-Y or UPDRS scales, which is following the results published by Oosterveld et al. (2020). We cannot exclude, however, that CSF NFL levels may be increased in cases of more pronounced axonal damage in PD dementia patients or late-stage PD subjects, who were not enrolled in this study to exclude major white matter damage. Gaetani et al. (2018), for example, found a positive correlation between CSF NFL levels and PD severity assessed in III motor part of UPDRS.

In our cohort of PD patients, the CSF NFL level correlated positively with age, which suggests that age also contributes to some degree to axonal degeneration. This positive correlation was not observed in healthy controls. This could be explained by the fact that all our control subjects were above 50 years old, so subjects with limited age range were involved in this study (*p* > 0.05).

A positive correlation between NFL and age in PD subjects was also found in other studies (Oosterveld et al., 2020). It is worth noting here that there is a high age dependency of NFL levels and NFL results should be always interpreted in the context of a control group matched for age. Due to this fact, our control subjects were matched for age (*p* > 0.05).

Further studies in a larger cohort of patients in different stages of the disease, using CSF and serum samples, should address the question of whether NFL levels may correlate with disease severity and disease progression.

### Limitations of the Study

Our study has some limitations. Firstly, PD was diagnosed based on clinical symptoms and not a neuropathological examination. Secondly, our study involved a limited number of PD patients, and only ones with early-stage PD. This could be the reason for the lack of correlations between CSF NFL and the markers of disease severity.

## Conclusion

This study indirectly demonstrates that axonal damage is present in PD in addition to neuronal loss. Interestingly, we observed white matter damage in non-demented PD patients. In the light of recent MRI studies that also report early white matter damage in PD which may even precede neuronal loss, our data may turn out to be of potential use in diagnosing early or even preclinical stages of the disease.

## Data Availability Statement

All datasets generated for this study are included in the article.

## Ethics Statement

The studies involving human participants were reviewed and approved by Ethics Commitee at the Medical University of Lublin, Poland. The patients/participants provided their written informed consent to participate in this study.

## Author Contributions

EP: conceptualization, data collection, investigation and writing—original draft. EP and KR: formal analysis and methodology. KR: software and writing—review and editing.

## Conflict of Interest

s The authors declare that the research was conducted in the absence of any commercial or financial relationships that could be construed as a potential conflict of interest.
